# Extracorporeal treatment of metforminassociated lactic acidosis in clinical practice: a retrospective cohort study

**DOI:** 10.1007/s00228-020-02857-5

**Published:** 2020-03-13

**Authors:** Inge R.F. van Berlo-van de Laar, Cornelis G. Vermeij, Marjo van den Elsen-Hutten, Arthur de Meijer, Katja Taxis, Frank G.A. Jansman

**Affiliations:** 1 Department of Clinical Pharmacy, Deventer Hospital, Nico Bolkesteinlaan 75, 7416 SE Deventer, P.O. Box 5001, 7400 GC Deventer, The Netherlands; 2 Department of Internal Medicine, Deventer Hospital, Nico Bolkesteinlaan 75, 7416 SE Deventer, The Netherlands; 3 Department of Research and Innovation, Deventer Hospital, Nico Bolkesteinlaan 75, 7416 SE Deventer, The Netherlands; 4 Department of Intensive Care, Deventer Hospital, Nico Bolkesteinlaan 75, 7416 SE Deventer, The Netherlands; 5 Unit of PharmacoTherapy, -Epidemiology &-Economics, Groningen Research Institute of Pharmacy (GRIP), University of Groningen, Antonius Deusinglaan 1, 9713 AV Groningen, The Netherlands

**Keywords:** Metformin, Lactic acidosis, Extracorporeal treatment, Renal impairment

## Abstract

**Purpose:**

To assess whether extracorporeal treatment (ECTR) improves outcome of patients with metformin-associated lactic acidosis (MALA) and to evaluate the clinical applicability of the Extracorporeal Treatments in Poisoning Workgroup (EXTRIP) criteria for starting ECTR in metformin poisoning.

**Methods:**

Patients with metformin serum concentrations above 2 mg/l who were admitted in the Deventer Teaching Hospital between January 2000 and July 2019 and complied with the definition of MALA (pH < 7.35 and lactate concentration > 5 mmol/l) were included. Mortality and clinical parameters of patients treated with ECTR or not were compared. In addition, treatment of MALA in clinical practice was verified against the criteria of EXTRIP.

**Results:**

Forty-two patients were included. Lactate (13.8 versus 10.5 mmol/l, *p* = 0.01), creatinine (575 versus 254 umol/l, *p* < 0.01)), metformin (29.4 versus 8.6 mg/l, *p* < 0.01) concentrations, and vasopressor requirement (72% versus 23%, *p* < 0.01) were significantly higher in the ECTR-group. Blood pH (7.05 versus 7.19, *p* = 0.03) and bicarbonate (6 versus 11 mmol/l, *p* < 0.01) were significantly lower. Mortality, length of hospital stay, and mechanical ventilation requirement were not statistically different. In 83% of patients, treatment of MALA was in accordance with the EXTRIP criteria.

**Conclusions:**

Although there was no statistical benefit in mortality shown from ECTR, ECTR might be lifesaving in MALA, considering the ECTR-group was significantly sicker than the non-ECTR-group.

The majority of patients were treated in line with the EXTRIP criteria. Severity of lactic acidosis and renal impairment were the main indications for initiating ECTR.

**Electronic supplementary material:**

The online version of this article (10.1007/s00228-020-02857-5) contains supplementary material, which is available to authorized users.

## Introduction

Metformin is the most commonly prescribed oral antidiabetic drug in non-insulin-dependent type 2 diabetes mellitus (NIDDM). Metformin inhibits gluconeogenesis, facilitates cellular glucose uptake, and decreases insulin resistance [1]. Metformin treatment is associated with a lower incidence of cardiovascular events and mortality in NIDDM [2]. Although metformin is considered to be a safe and well tolerated drug, its use may rarely be complicated by lactic acidosis [1, 3–6]. The most widely accepted mechanism how metformin causes hyperlactatemia and metabolic acidosis is by partial inhibition of oxidative phosphorylation complex 1 of the mitochondrial electron transport chain. Another possible mechanism in which metformin may elevate plasma lactate levels is through inhibition of pyruvate carboxylase which results in both accelerated lactate production and reduced lactate metabolism [1, 3–5]. There appears to be a clear relationship between metformin accumulation and lactic acidosis, although some authors have pointed out that several such patients had other confounding risk factors for lactic acidosis [3–5, 7].

Metformin-associated lactic acidosis (MALA) is a serious adverse event with a high mortality rate of up to 50% [1, 4]. The incidence of MALA varies from 0 to 138 per 100.000 patient years and may increase in the coming years due to the increase in the number of type 2 diabetes mellitus patients and the use of metformin [4, 6, 8, 9]. Several studies suggest that starting timely treatment might reduce MALA-related morbidity and mortality [8–14]. Extracorporeal treatments (ECTRs) may be necessary to remove metformin, clear lactate, and correct acid-base abnormalities [1, 11]. Calello et al. [1] formulated specific recommendations for starting ECTR in metformin poisoning based on a systematic literature search: the Extracorporeal Treatments in Poisoning Workgroup (EXTRIP) criteria [15], which have been included in the treatment guidelines for metformin intoxication by the Dutch Poisons Information Centre (DPIC) [16]. However, the evidence levels of the EXTRIP criteria are low and their validity in clinical practice has not been assessed yet. We therefore evaluated the treatment of MALA patients in clinical practice. The aim of this study was firstly to assess whether ECTR improves outcome of MALA patients. Secondly, we aimed to evaluate whether the EXTRIP criteria for starting ECTR in MALA are applicable in clinical practice, i.e., to what extent patients who received ECTR and those who did not fulfill the EXTRIP criteria for starting ECTR [1].

## Methods

A retrospective single-center cohort study was conducted at the Deventer Teaching Hospital in the Netherlands. Laboratory data were searched for patients who had their metformin serum concentrations measured between January 2000 and July 2019. In these patients, serum metformin concentration measurement had been requested because of a clinical suspicion of MALA, based on documented metformin use and concurrent illness leading to an emergency department visit. In the Deventer Teaching Hospital, the metformin assay is routinely available 24 h a day. Results are available for clinical decisions within 4 h. Patients were included if they met the MALA definition: pH < 7.35 and lactate > 5.0 mmol/l in association with metformin exposure [1]. Only patients with serum metformin concentrations above the lower limit of quantification of our analysis method, i.e., 2 mg/l, were included. The following patient data were extracted from the medical records: age, gender, admission diagnosis, ECTR treatment (or not), reasons for initiating ECTR (or not), decreased consciousness, vasopressor requirement, mechanical ventilation requirement, length of hospital stay, mortality (defined as in-hospital mortality), and laboratory results on admission: serum concentrations of creatinine, lactate, bicarbonate and metformin, and blood pH. In the Deventer Teaching Hospital, ECTR is readily and unrestrictedly available for treatment of MALA patients.

Patients were divided into an ECTR and non-ECTR group, and the concentrations of lactate, creatinine, bicarbonate and metformin, blood pH, decreased consciousness, vasopressor and mechanical ventilation requirement, length of hospital stay, and mortality were compared. In case of normal distribution of continuous data, the independent sample *t* test was used. The non-parametric Mann-Whitney test was used for not normally distributed and ordinal data. The Chi square test was used to compare nominal data between groups. In all tests, a *p* value < 0.05 was considered statistically significant. Data analysis was performed with SPSS version 24.0.

In the ECTR and non-ECTR group, we assessed whether patients met the EXTRIP criteria for starting ECTR depicted in Table 1. Impaired kidney function is defined by the EXTRIP nephrology sub-committee as (1) advanced stage G3b, G4, or G5 chronic kidney disease (i.e., eGFR < 45 mL/min/1.73 m^2^), (2) kidney disease: Improving Global Outcomes (KDIGO) stage 2 or 3 acute kidney injuries, (3) in the absence of a baseline serum creatinine, 176 μmol/L in adults and 132 μmol/L in elderly/low muscle mass patients, and (4) the presence of oligo/anuria regardless of serum creatinine concentration.

**Table 1:** EXTRIP criteria for starting ECTR in metformin poisoning [1]

Indications	
ECTR is recommended if: • Lactate concentration greater than 20 mmol/l • Blood pH less than or equal to 7.0 • Standard therapy (supportive measures, bicarbonate, etc.) fails ECTR is suggested if: • Lactate concentration is 15–20 mmol/l • Blood pH 7.0–7.1 Comorbid conditions that lower the threshold for initiating ECTR: • Impaired kidney function • Shock • Decreased level of consciousness • Liver failure	

*EXTRIP* Extracorporeal Treatments in Poisoning Workgroup

*ECTR* Extracorporeal treatment

In those patients who were not treated according to the EXTRIP criteria, the reasons for initiating ECTR or not were evaluated.

## Results

In our hospital pharmacy laboratory database, we identified 160 patients who had serum metformin concentrations measured. Of these, 42 patients met the inclusion criteria of MALA and were included in the study. Forty patients (95%) had renal impairment on admission and 29 patients (69%) were treated with ECTR. ECTR was conducted in the intensive care unit. ECTR modalities used were continuous veno venous hemofiltration (CVVH) (19 patients), hemodialysis (HD) (7 patients), or a sequential combination of CVVH and HD (3 patients). The patient characteristics and the results of the comparison between the ECTR and non-ECTR groups are listed in Table 2. The main admission diagnoses were dehydration, sepsis, shock, and myocardial infarction. Detailed information of the patient characteristics per patient is given in Online Resource 1 (ECTR-group) and Online Resource 2 (non-ECTR-group).

**Table 2:** Results: patient characteristics and comparison clinical parameters ECTR versus non-ECTR group

Patient characteristics	ECTR *N* = 29 Mean ± sd (range)	Non-ECTR *N* = 13 Mean ± sd (range)	Statistical analysis
Gender	6 M 23 F	5 M 8 F	
Age (years)	71 ± 9 (52–87)	77 ± 11 (58–89)	
pH	7.05 ± 0.18 (6.61–7.34)	7.19 ± 0.18 (6.85–7.33)	*p* = 0.027
Lactate (mmol/l)	13.8 ± 4.9 (5.8–23.2)	10.5 ± 2.8 (6.7–18)	*p* = 0.033
Bicarbonate (mmol/l)	6 ± 3 (2–13)	11 ± 4 (2–17)	*p* < 0.01
Metformin concentration (mg/l)	29.4 ± 20.3 (2.3–100)	8.6 ± 11.2 (2.2–37)	*p* < 0.01
Creatinine (umol/l)	575 ± 268 (113–1039)	254 ± 192 (70–720)	*p* < 0.01
Decreased consciousness N (%)	9 (31%)	3 (23%)	*p* = 0.699
Vassopressor requirement N (%)	21 (72%)	3 (23%)	*p* < 0.01
Mechanical ventilation requirement N (%)	6 (21%)	1 (8%)	*p* = 0.296
Length of stay (days)	17.3 ± 23.6 (2–120)	7.8 ± 9.0 (1–32)	*p* = 0.067
Mortality N (%)	11 (38%)	6 (46%)	*p* = 0.616

*ECTR* Extracorporeal Treatment

Thirty-five of the 42 (83%) patients were treated in line with the EXTRIP criteria.

Of the 29 patients in the ECTR-group, 28 (97%) fulfilled the EXTRIP criteria to receive ECTR. Clinical reasons for starting ECTR in these patients were severe metabolic acidosis, renal failure, hyperkalaemia, and high metformin concentrations. Ninety-seven percent of the ECTR group met the criterion of impaired renal function of Calello et al. [1] in which the threshold for initiating ECTR could be lowered. One patient (patient no. 27, Online Resource 1) did not fulfill the EXTRIP criteria. This patient was admitted because of an intentional overdose and did not meet the criterion of impaired renal function of Calello et al. [1]. ECTR was started because of the combination high serum metformin concentration and lactic acidosis in order to eliminate metformin and to correct the acidosis.

Of the 13 patients in the non-ECTR group, in 7 (54%) of the patients treatment, (non-ECTR) was in line with the EXTRIP criteria. One patient (patient no. 6, Online Resource 2) did not fulfill the EXTRIP criteria for starting ECTR, and in 6 patients, ECTR was not necessary because they recovered after starting supportive care.

For the other 6 (46%) patients, ECTR should have been considered according to the EXTRIP criteria. Supportive care was started in these patients but they died shortly after the start of the treatment. Four patients died within 1 day from cardiac arrest. In one patient, a conservative policy was started because of the very bad prognosis due to comorbidity and she died 1 day after admittance. One patient (patient no.10, Online Resource 2) did not recover with supportive care and died 1 month after admission probably from sepsis. There were no data available in this patients’ medical record whether ECTR was considered.

## Discussion

This retrospective cohort study shows a lower but not statistically different mortality in MALA patients treated with ECTR compared to those who were not. The overall mortality of 40% in our study is in line with the mortality reported in previous studies, ranging from 20 to 50% [7, 8, 12–14, 17–21]. Blood pH, lactate, creatinine, and serum metformin concentration in the ECTR group in this study are similar to that reported in the literature [9, 13, 20–22]. The significantly higher lactate and creatinine concentrations in the ECTR group compared to the non-ECTR group have also been reported in other studies [9, 12, 19].

Patients in the ECTR group were sicker than patients in the non-ECTR group considering the degree of lactic acidosis, kidney function, and vasopressor requirement while having a lower but not statistically different, mortality. As hyperlactatemia in general and in MALA patients is associated with increased mortality [8, 11, 23–25], this at least comparable outcome suggests there might be a benefit for ECTR. This is also suggested by Peters et al. [19]. Our study was probably underpowered to show a statistical difference. We also compared the length of hospital stay (17.3 versus 7.8 days, *p* = 0.067), but in this study, this parameter is less suitable as outcome measure compared to mortality because of the large range in the ECTR group (2–120 days) and the high percentage patients who died within 1–2 days in the non-ECTR group.

To evaluate whether the EXTRIP criteria for initiating ECTR in patients with MALA are applicable in clinical practice, we compared the indications for starting ECTR in this study with the recommendations of Calello et al. [1]. Overall, 83% of our patients were treated in line with the EXTRIP criteria. In the ECTR group, 97% and in the non-ECTR group 54% of the patients fulfilled the EXTRIP criteria. Severity of lactic acidosis and kidney function were the main indications for initiating ECTR in this study. This is also shown in the EXTRIP criteria [1] and the study of Corcia et al. [9]. Moreover, in accordance with Corchia et al. [9], we identified hyperkalaemia as a reason for starting ECTR. In contrast, hemodynamic instability and shock, as proposed by Corcia et al. [9] and EXTRIP [1, 15] for initiating ECTR, were not recorded in the patients’ medical records in this study. Calello et al. [1] have not formulated a threshold for metformin serum concentration because at the time of formulation of these recommendations, there was much uncertainty regarding the value of metformin concentrations in relation to the prognosis and the limited availability of the metformin assays. Some studies have shown a correlation between metformin concentration and mortality [8, 9, 20] while others have not [17, 21, 25, 26]. Despite the uncertainty concerning its prognostic value, measuring metformin serum concentrations could be of diagnostic value in MALA and may assist in its management [9, 22, 26]. However, establishing a specific threshold for metformin serum concentrations is not possible based on the results of this study.

The EXTRIP criteria include lowering thresholds of pH and lactate for initiating ECTR in impaired kidney function, shock, decreased level of consciousness, and liver failure but this is not quantified. The majority of the ECTR group in this study had impaired renal function, and the mean pH and lactate concentration were 7.05 and 13.8 mmol/l respectively. In clinical practice, comorbidity is common, and it is not always clear whether there is metformin accumulation, showing the heterogeneity regarding the EXTRIP criteria and real-life scenarios. Because of this heterogeneity, formulating more concise criteria for initiating ECTR in MALA patients is very difficult. The main reasons for not initiating ECTR in this study were recovery after starting supportive care or death shortly after admission. Six patients who met the EXTRIP criteria were not treated with ECTR and died. At the time of admission of these patients, the EXTRIP criteria were not implemented in our hospital. Four out of these six patients died within 1 day from cardiac arrest and there was no renal indication for starting ECTR. Additionally, in the non-ECTR group, 54% of patients had serum metformin concentrations lower than 5 mg/l which is in line with the ‘normal’ value of serum metformin concentrations in therapeutic use [4, 27]. Therefore, it is debatable whether metformin was the cause of MALA in these patients. Lalau et al. [4] suggested adding serum metformin concentration higher than 5 mg/l as criterion to MALA to distinguish it from metformin unrelated lactic acidosis (MULA). However, we used the definition of MALA pH < 7.35 and lactate >5 mmol/l in association with metformin exposure as formulated by Calello et al. [1] because we wanted to evaluate Calello’s recommendations in clinical practice. In addition, we validated metformin exposure by only including patients with verifiable serum metformin concentrations to avoid discussion about metformin exposure.

The present study is one of the largest cohort studies regarding the management of MALA. The strength of our study is that metformin concentrations, lactate, blood pH, and kidney function were measured simultaneously on admission and during subsequent treatment. Furthermore, only patients with verified metformin serum concentrations were included. Lalau et al. [4] presented the lack of these combined data as major methodological flaw in most studies on MALA. However, we did not measure metformin concentrations in erythrocytes, which probably better reflects metformin tissue effects, and we have no information on last intake so we cannot refer to peak versus trough concentrations [4]. A limitation of our study is that other causes of lactic acidosis were not ruled out which could have influenced the mortality in this study. Other limitations include the retrospective and monocentric design and selection bias. We selected patients based on serum metformin concentration measurement. MALA patients without serum metformin concentration measurement could have been missed. Finally, as presented in the EXTRIP guidelines, metformin and lactate clearance are lower with continuous renal replacement therapy (CRRT) than with intermittent HD. As such, the predominant use of CVVH in our study might have weakened the results in favor for ECTR.

For clinical practice, we recommend that clinicians be alert to MALA in the emergency department when patients are admitted with lactic acidosis in combination with metformin use. ECTR might be lifesaving in the treatment of MALA and should therefore be considered at an early stage. The EXTRIP-criteria are a good starting point for the decision to start ECTR but each individual patient needs to be evaluated separately. Severity of lactic acidosis and renal impairment are the main indications for initiating ECTR. Knowledge of the metformin concentrations may be a valuable additional parameter for the diagnosis and management of MALA. Therefore, we recommend implementing metformin assays as routine investigation with 24-h availability in hospitals treating MALA patients.

## Conclusion

Although there was no statistical difference in mortality between the treatment with or without ECTR, ECTR might be lifesaving in treating MALA. Patients in the ECTR group were sicker compared to the non-ECTR group considering the degree of lactic acidosis, kidney function, and vasopressor requirement and had at least a comparable mortality. In 83% of the patients, treatment was in line with the EXTRIP criteria. Severity of lactic acidosis and renal impairment were the main indications for initiating ECTR. Measuring serum metformin concentrations may assist in the diagnosis and management of MALA.

## Electronic supplementary material

ESM 1 (PDF 64 kb)

ESM 2 (PDF 34 kb)

## References

[1] Calello DP, Liu KD, Wiegand TJ, Roberts DM, Lavergne V, Gosselin S, Hoffman RS, Nolin TD, Ghannoum M, Extracorporeal Treatments in Poisoning Workgroup (2015). Extracorporeal treatment for metformin poisoning: systematic review and recommendations from the extracorporeal treatments in poisoning workgroup. Crit Care Med.

[2] UK Prospective Diabetes Study Group (1998). Effect of intensive blood-glucose control with metformin on complications in overweight patients with type 2 diabetes (UKPDS 34). Lancet.

[3] Wang George Sam, Hoyte Christopher (2018). Review of Biguanide (Metformin) Toxicity. Journal of Intensive Care Medicine.

[4] Lalau JD, Kajbaf F, Protti A, Christensen MM, De Broe ME, Wiernsperger N (2017). Metformin-associated lactic acidosis (MALA): moving towards a new paradigm. Diabetes Obes Metab.

[5] DeFronzo R, Fleming GA, Chen K, Bicsak TA (2016). Metformin-associated lactic acidosis: current perspectives on causes and risks. Metabolism.

[6] Berlo van-Laar van de IRF, Vermeij CG, Doorenbos CJ (2011). Metformin associated lactic acidosis: incidence and clinical correlation with metformin serum concentration measurements. J Clin Pharm Ther.

[7] Kuan Isabelle H. S., Savage Ruth L., Duffull Stephen B., Walker Robert J., Wright Daniel F. B. (2019). The Association between Metformin Therapy and Lactic Acidosis. Drug Safety.

[8] Boucaud-Maitre D, Ropers J, Porokhov B, Altman JJ, Bouhanick B, Doucet J (2016). Lactic acidosis: relationship between metformin levels, lactate concentration and mortality. Diabet Med.

[9] Corchia A, Wynckel A, Journet J, Frances JM, Skandrani N, Lautrette A et al (2019) Metformin-related lactic acidosis with acute kidney injury: results of a French observational multicentre study. Clin Toxicol 1–8 10.1080/15563650.2019.1648816 31387415

[10] Semely D, Bennett E, Vallejo C, Saint-Marcoux F, Merle L, Nouaille Y (2016). Can an early diagnostic procedure of metformin-associated lactic acidosis in an emergency unit reduce mortality?. Therap.

[11] Jung B, Martinez M, Claessens YE, Darmon M, Klouche K, Lautrette A (2019). Diagnosis and management of metabolic acidosis: guidelines from a French expert panel. Ann Intensive Care.

[12] Moioli A, Maresca B, Manzione A, Napoletano AM, Coclite D, Pirozzi N (2016). Metformin associated lactic acidosis (MALA): clinical profiling and management. J Nephrol.

[13] Mariano F, Pozzato M, Inguaggiato P, Guarena C, Turello E, Manes M, David P, Berutti S, Consiglio V, Amore A, Campo A, Marino A, Berto M, Carpani P, Calabrese G, Gherzi M, Stramignoni E, Martina G, Serra A, Comune L, Roscini E, Marciello A, Todini V, Vio P, Filiberti O, Boero R, Cantaluppi V (2017). Metformin-associated lactic acidosis undergoing renal replacement therapy in intensive care units: a five million population-based study in the north-west of Italy. Blood Purif.

[14] Angioi A, Cabiddu G, Conti M, Pili G, Atzeni A, Matta V, Cao R, Floris M, Songini M, Mulas MF, Rosner M, Pani A (2018). Metformin associated lactic acidosis: a case series of 28 patients treated with sustained low-efficiency dialysis (SLED) and long term follow up. BMC Nephrol.

[15] EXTRIP-workgroup. Metformin. Available from: https://www.extrip-workgroup.org/metformin. Accessed 12 Feb 2019

[16] UMC Utrecht Nationaal Vergiftigingen Informatie Centrum Metformine. UMC Utrecht Nationaal Vergiftigingen Informatie Centrum, Utrecht. Available from: https://www.vergiftigingen.info/f?p=300:STOFMONOGRAFIE:10568776339678::NO. Accessed 1 June 2019

[17] Seidowsky A, Nseir S, Houdret N, Fourrier F (2009). Metformin-associated lactic acidosis: a prognostic and therapeutic study. Crit Care Med.

[18] Greco P, Reolisti G, Maggiore U, Feriolo E, Fani F, Loratelli C (2019). Sustained low-efficiency dialysis for metformin associated lactic acidosis in patients with acute kidney injury. J Nephrol.

[19] Peters N, Jay N, Barraud D, Cravoisy A, Nace L, Bollaer PE (2008). Metformin-associated lactic acidosis in an intensive care unit. Crit Care.

[20] Friesecke S, Abel P, Roser M, Felix SB, Runge S (2010). Outcome of severe lactic acidosis associated with metformin accumulation. Crit Care.

[21] Vecchio S, Giampretti A, Petrolini M, Lonati D, Protti A, Papa P (2014). Metformin accumulation: lactic acidosis and high plasmatic metformin levels in a retrospective case series of 66 patients on chronic therapy. Clin Toxicol.

[22] Duong JK, Furlong TJ, Roberts DM, Graham GG, Greenfield JR, Williams KM, Day RO (2013). The role of metformin in metformin-associated lactic acidosis (MALA): case series and formulation of a model of pathogenesis. Drug Saf.

[23] van den DPA N, Brouwers MCG, Stassen PM (2017). Prognostic value of plasma lactate levels in a retrospective cohort presenting at a university hospital emergency department. BMJ Open.

[24] Kjelland CB, Djogovic D (2010). The role of serum lactate in the acute care setting. J Intensive Care Med.

[25] Yeh HC, Ting IW, Tsai CW, Wu JY, Kuo CC (2017). Serum lactate level and mortality in metformin associated lactic acidosis requiring renal replacement therapy: a systematic review of case reports and case series. BMC Nephrol.

[26] Kajbaf F, Lalau JD (2013). The prognostic value of blood pH and lactate and metformin concentrations in severe metformin-associated lactic acidosis. BMC Pharmacol Toxicol.

[27] Kajbaf F, De Broe ME, Lalau JD (2016). Therapeutic concentrations of metformin: a systematic review. Clin Pharmacokinet.

