# Supplementary material for: Extracorporeal treatment of metforminassociated lactic acidosis in clinical practice: a retrospective cohort study
Source: Eur J Clin Pharmacol. 2020 Mar 13;76(6):815–20. doi: 10.1007/s00228-020-02857-5 (PMC7239820; doi:10.1007/s00228-020-02857-5)
Supplement: Supplementary file 1 — (PDF 64 kb) [file 228_2020_2857_MOESM1_ESM.pdf]

Article title: Extracorporeal treatment of Metformin Associated Lactic Acidosis in clinical practice: a retrospective cohort study

Journal name: European Journal of Clinical Pharmacology

Author names: Inge R.F. van Berlo-van de Laar, Cornelis G. Vermeij, Marjo van den Elsen- Hutten, Arthur de Meijer, Katja Taxis, Frank G.A. Jansman

Affiliation corresponding author: Department of Clinical Pharmacy, Deventer Hospital, Nico Bolkesteinlaan 75, 7416 SE Deventer, The Netherlands

Email address: i.vanberlo-vandelaar@dz.nl

Patient characteristics ECTR group

Nr	Sex	Age (years)	pH	Lactate (mmol/l)	Bic (mmol/l)	Metformin (mg/l)	Creatinine (umol/l)	Diagnosis	DC	VR	MVR	LoS (days)	Outcome	Reason ECTR
1	F	67	7.05	19.0	8	2.9	478	Hemorrhagic shock	Y	N	N	34	D	Renal failure
2	F	66	7.12	13.5	7	19.4	640	Dehydration	Y	Y	N	120	S	Severe metabolic acidosis and renal failure with metformin use
3	F	75	7.03	16.5	7	5.0	266	Myocardial infarction	Y	Y	N	2	D	Severe metabolic acidosis and renal failure with metformin use
4	F	75	7.22	5.8	12	32	813	Myocardial infarction	N	N	N	18	S	Renal failure
5	M	58	6.77	23	2	38	1039	Dehydration	N	Y	Y	19	S	Severe metabolic acidosis and renal failure with metformin use
6	F	72	7.15	14.5	8	20	814	Renal failure	N	N	N	38	S	Severe metabolic acidosis and renal failure with metformin use
7	F	77	7.18	11.1	7	25.4	960	Dehydration	N	N	N	22	S	Renal failure, hyperkalaemia, MALA
8	F	68	7.07	9.9	10	22	706	Dehydration	N	N	N	2	S	Renal failure, hyperkalaemia

9	F	78	6.94	20.3	4	44	612	Dehydration	N	Y	N	2	S	Severe metabolic acidosis and renal failure with metformin use
10	F	62	6.9	13.8	2	34	590	Dehydration	Y	Y	N	14	S	Severe metabolic acidosis and renal failure with metformin use
11	F	82	6.82	23.2	2	47	691	Dehydration	N	Y	N	15	S	Severe metabolic acidosis and high metformin serum concentration
12	F	56	7.12	8.8	7	31	882	Dehydration	Y	Y	N	7	S	Severe metabolic acidosis and renal failure with metformin use, hyperkalaemia
13	F	67	7.25	6.6	12	22.8	519	Pneumonia	N	Y	N	18	D	Lactic acidosis and high metformin serum concentration
14	F	62	6.61	15.6	4	45	808	Septic shock	Y	Y	Y	2	D	Failure supportive care, severe metabolic acidosis and high metformin serum concentration
15	F	76	7.12	8.0	3	27.5	669	Urosepsis	N	Y	N	14	S	Failure supportive care, high metformin serum concentration
16	F	72	6.82	11.2	4	45	1004	Dehydration	N	Y	N	2	D	Severe metabolic acidosis and renal failure with metformin use
17	M	82	7.12	8.8	8	4.1	414	Urosepsis	N	N	N	25	S	Renal failure, hyperkalaemia
18	M	87	7.34	11.7	9	8.2	263	Urosepsis	N	Y	N	2	D	MALA
19	F	79	6.94	13.5	2	46	642	Dehydration	N	Y	N	8	S	Failure supportive care, severe metabolic acidosis
20	F	78	7.22	14.3	8	12.6	147	Heart failure	N	N	N	11	D	MALA
21	F	70	6.98	12.8	8	42.4	419	Septic shock	Y	Y	Y	5	D	Severe metabolic acidosis, high metformin serum concentration
22	F	69	7.22	19	7	23.4	523	Dehydration	N	Y	N	9	S	Failure supportive care, lactic acidosis, renal failure,

														high metformin serum concentration
23	F	82	6.66	10.6	2	44.5	574	Septic shock	Y	Y	Y	60	S	Renal failure, severe metabolic acidosis
24	F	72	7.14	6.5	8	30.4	841	Sepsis	N	Y	Y	17	D	Failure supportive care
25	F	72	7.16	14	4	2.3	140	Heart failure	Y	N	N	11	D	MALA
26	M	58	7.00	21.6	6	100	208	Intentional overdose	N	Y	Y	10	S	Severe metformin intoxication, lactic acidosis
27	M	52	7.26	11.4	13	50	113	Intentional overdose	N	Y	N	5	S	Severe metformin intoxication, lactic acidosis
28	M	69	7.17	16	5	4.6	255	Hemorrhagic shock	N	Y	N	3	D	Renal failure, MALA
29	F	65	6.97	18	4	23.3	645	Dehydration	N	Y	N	8	S	Renal failure, hyperkalaemia, MALA

DC = decreased conciousness; Y= yes, N = no, U = unknown
VR = Vassopressor requirement: Y= yes, N = no
MVR = mechanical ventilation requirement: Y= yes, N = no
LoS = Length of Stay
Outcome: D = died, S = survived
MALA = metformin associated lactic acidosis
