# Supplementary material for: Extracorporeal treatment of metforminassociated lactic acidosis in clinical practice: a retrospective cohort study
Source: Eur J Clin Pharmacol. 2020 Mar 13;76(6):815–20. doi: 10.1007/s00228-020-02857-5 (PMC7239820; doi:10.1007/s00228-020-02857-5)
Supplement: Supplementary file 2 — (PDF 34 kb) [file 228_2020_2857_MOESM2_ESM.pdf]

Article title: Extracorporeal treatment of Metformin Associated Lactic Acidosis in clinical practice: a retrospective cohort study

Journal name: European Journal of Clinical Pharmacology

Author names: Inge R.F. van Berlo-van de Laar, Cornelis G. Vermeij, Marjo van den Elsen- Hutten, Arthur de Meijer, Katja Taxis, Frank G.A. Jansman

Affiliation corresponding author: Department of Clinical Pharmacy, Deventer Hospital, Nico Bolkesteinlaan 75, 7416 SE Deventer, The Netherlands

Email address: i.vanberlo-vandelaar@dz.nl

Patientcharacteristics non-ECTR group

Nr	Sex	Age (years)	pH	Lactate (mmol/l)	Bic (mmol/l)	Metformin (mg/l)	Creatinine (umol/l)	Diagnosis	DC	VR	MVR	LoS (days)	Outcome	Reason ECTR
1	F	78	6.85	10.2	2	4	547	Dehydration	N	N	N	1	D	Supportive care but cardiac arrest and passed away the same night
2	F	87	7.24	9.4	10	2.5	146	Cardiogenic shock, myocardial infarction	N	N	N	2	D	Supportive care but passed away within 1 day after admittance
3	F	85	7.33	8.8	13	6.8	257	Hypovolemic shock by bleeding	N	N	N	10	S	Recovery with supportive care
4	F	58	7.04	12.6	7	37	720	Sepsis	N	N	N	6	S	Recovery with supportive care
5	M	89	7.3	9.4	12	6.1	136	Hypovolemic shock by bleeding	N	N	N	12	S	Recovery with supportive care
6	M	63	7.31	9	14	3	73	MALA	N	N	N	3	S	Recovery with supportive care
7	F	80	7.14	13.3	12	3	366	Pneumosepsis	Y	N	N	2	D	No recovery with supportive care, conservative policy because of bad prognosis, passed away within 1 day after admittance

8	M	58	7.08	8.8	11	7.9	207	Myocardial infarction	N	Y	Y	1	D	No recovery with supportive care, passed away within 1 day after admittance from cardiac arrest
9	F	77	7.32	10.4	11	29.3	70	MALA	Y	N	N	3	S	Recovery with supportive care
10	F	88	7.3	11	14	2.3	126	Pneumonia, 3rd grade AV-block	N	Y	N	32	D	No recovery with supportive care, passed away probably from sepsis
11	M	83	7.33	9.3	7	2.4	158	Cardiogenic shock	U	N	N	17	S	Recovery with supportive care
12	F	78	7.33	6.7	17	2.2	200	Dehydration	N	N	N	12	S	Recovery with supportive care
13	M	77	6.86	18	6	5.5	301	Myocardial infarction	Y	Y	N	1	D	Passed away after cardiopulmonary resuscitation (CPR)

DC = decreased conciousness; Y= yes, N = no, U = unknown
VR = Vassopressor requirement: Y= yes, N = no
MVR = mechanical ventilation requirement: Y= yes, N = no
LoS = Length of Stay
Outcome: D = died, S = survived
MALA = metformin associated lactic acidosis
